# Supplementary material for: Engineering the Active Site Lid Dynamics to Improve the Catalytic Efficiency of Yeast Cytosine Deaminase
Source: Int J Mol Sci. 2023 Apr 1;24(7):6592. doi: 10.3390/ijms24076592 (PMC10095239; doi:10.3390/ijms24076592)
Supplement: Supplementary file 1 [file ijms-24-06592-s001.zip › ijms-2300210-supplementary.pdf]

Engineering the Active Site Lid Dynamics to Improve the Catalytic Efficiency of Yeast Cytosine Deaminase

Hanzhong Deng,[1,2,3,⊥] Mingming Qin,[1,2,⊥] Zhijun Liu,[4,⊥] Ying Yang[1,2], Yefei Wang,[1,2,*] and Lishan Yao[1,2,*]

[1]Qingdao New Energy Shandong Laboratory, Qingdao Institute of Bioenergy and Bioprocess Technology, Chinese Academy of Sciences, Qingdao, 266101, China

[2]Shandong Energy Institute, Qingdao, 266101, China

[3]University of Chinese Academy of Sciences, Beijing, 100049, China

[4]National Facility for Protein Science, Shanghai Advanced Research Institute, Chinese Academy of Sciences, Shanghai, 201210, China

Supporting Information

Table S1. Backbone amide H/D exchange rates of residues in the wt-yCD, yCD-RQ, yCD-RQ-1/8SAH, and yCD-RQ-1/4SAH complexed with the TSA*.

Res	WT	RQ	1/8 SAH	1/4 SAH
8	F	F	F	F
10	F	F	F	F
11	F	F	F	F
12	F	F	F	F
13	F	F	F	F
14	1.07	0.94	0.88	1.06
15	S	S	S	S
16	S	S	S	S
17	S	S	S	S
18	S	S	S	S
19	S	S	S	S
20	S	S	S	S
21	S	S	S	S
22	S	S	S	S
23	S	S	S	S
24	S	S	S	S
25	S	S	S	S
26	S	S	S	S
28	0.04	0.06	0.04	0.05
29	F	F	F	F
30	S	S	S	S
31	S	S	S	S
33	S	S	S	S
34	S	S	S	S
35	S	S	S	S
36	S	S	S	S
37	S	S	S	S
38	S	S	S	S
39	S	S	S	S
40	1.15	1.27	1.34	1.51
41	F	F	F	F
42	F	F	F	F
43	0.44	0.43	0.35	0.43
44	0.92	0.85	0.81	0.99
46	S	S	S	S
47	S	S	S	S
48	6.35	5.26	5.16	6.36
49	S	S	S	S
50	F	F	F	F

52	S	S	S	S
53	S	S	S	S
54	S	S	S	S
55	1.38	1.20	1.61	1.70
56	0.35	0.42	0.36	0.44
57	S	S	S	S
58	S	S	S	S
59	0.33	1.21	1.72	2.25
60	S	S	S	S
62	S	S	S	S
63	4.29	F	F	F
65	S	S	S	S
66	S	S	S	S
67	S	S	S	S
68	S	S	S	S
69	S	S	S	S
70	S	S	S	S
71	S	S	S	S
72	0.25	0.68	0.71	0.82
73	F	F	F	F
74	0.25	1.41	1.86	2.23
75	10.02	F	F	F
76	F	F	F	F
77	F	F	F	F
78	3.06	F	F	F
79	0.17	1.30	1.74	1.98
80	0.17	1.54	1.95	2.47
81	F	F	F	F
82	S	S	S	S
83	S	S	S	S
84	S	S	S	S
85	S	S	S	S
86	S	S	S	S
87	S	S	S	S
89	S	S	S	S
91	0.06	0.20	0.11	0.37
93	S	S	S	S
94	S	S	S	S
95	S	S	S	S
96	S	S	S	S
97	S	S	S	S
98	S	S	S	S

99	S	S	S	S
100	S	S	S	S
101	S	S	S	S
102	S	S	S	S
103	S	S	S	S
105	S	S	S	S
106	S	S	S	S
107	S	S	S	S
108	S	S	S	S
109	S	S	S	S
110	S	S	S	S
111	0.93	0.66	0.49	0.58
112	S	S	S	S
114	0.21	0.15	0.09	0.13
115	F	F	F	F
116	F	F	F	F
118	F	F	F	F
119	F	F	F	F
120	F	F	F	F
121	F	F	F	F
122	1.26	0.81	0.61	0.78
123	2.76	1.85	1.71	2.21
124	F	F	F	F
126	F	F	F	F
128	0.76	0.68	0.60	0.63
129	F	F	F	F
130	S	S	S	S
131	F	F	F	F
132	S	S	S	S
133	S	S	S	S
134	0.39	0.35	0.28	0.34
135	F	F	F	F
136	F	F	F	F
137	0.26	0.25	0.21	0.26
138	1.75	1.36	1.63	1.79
139	3.60	3.53	4.38	4.93
140	1.22	1.08	1.12	1.35
141	S	S	S	S
142	S	S	S	S
143	0.38	0.42	0.38	0.46
144	S	S	S	S
145	S	S	S	S

146	S	S	S	S
147	S	S	S	S
148	S	S	S	S
150	F	F	F	F
151	5.10	7.89	10.71	F
152	S	S	S	S
153	0.05	0.37	0.40	0.61
154	0.78	2.55	3.58	7.16
155	1.38	8.77	10.66	F
156	0.24	1.51	2.31	3.87
157	1.21	5.31	7.82	10.08
158	0.10	F	F	F

*F means that the exchange rate is faster than the detection limit (15 h^{-1}), and S means that the exchange does not occur during the detection period (6 h).

Table S2. Data collection and refinement statistics.[a]

**Protein**	yCD-RQ	yCD-RQ-1/8SAH	yCD-RQ-1/8SAH
**Ligand**	Apo	Apo	4-[S]-hydroxyl-3,4 -dihydropyrimidine
**PDB Entry**	**8I3N**	**8I3O**	**8I3P**
**Data collection**			
Space group	*P 1 21 1*	*P 1 21 1*	*P 1 21 1*
Unit cell dimensions			
a, b, c (Å)	61.319 64.273 83.092	61.669 64.47 83.092	49.175 68.9 49.424
α, β, γ (°)	90 97.69 90	90 97.965 90	90 101.581 90
Multiplicity	6.3 (6.0)	3.7 (3.8)	2.5 (2.5)
Completeness (%)	98.68 (98.00)	96.61 (97.60)	85.99 (89.17)
I/σ_I	16.12 (7.64)	12.31 (2.97)	19.03 (5.39)
R_{merge}	0.079 (0.213)	0.069(0.584)	0.040 (0.185)
Wilson B factor ($Å^2$)	23.92	22.35	10.80
$CC_{1/2}$	0.997 (0.986)	0.996 (0.873)	0.998 (0.948)
**Refinement**			
Resolution range (Å)	34.67 - 2.00 (2.07 - 2.00)	34.68 - 1.73 (1.792 - 1.73)	33.41 - 1.3 (1.346 - 1.3)
NO. reflections	42979 (4270)	65362 (6554)	68215 (7046)
R_{work}/R_{free}	0.1739/0.2071	0.2029/0.2170	0.1572/0.1789
Number of non-hydrogen atoms	5099	5134	2886
Macromolecules	4586	4586	2511
Ligands/ions	4	4	26
Solvent	509	544	349
Average B-factor	26.62	28.14	16.24
Macromolecules	25.80	27.06	14.92
Ligands	17.65	23.33	15.73
Solvent	34.13	37.28	25.83
r.m.s.d. for ideal value			
Bond length (Å)	0.003	0.006	0.016
Bond angle (°)	0.51	0.58	1.43
Ramachandran plot			
Favored/allowed/outliers (%)	98.66/1.34/0	99.33/0.67/0	98.47/1.53/0

[a] Values for the highest resolution shell are given in parentheses.

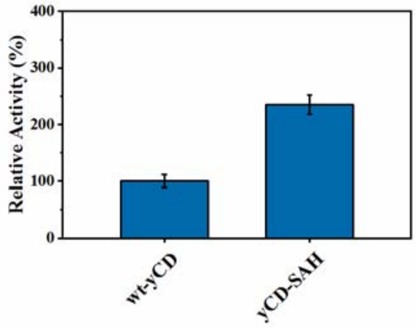

Figure S1. wt-yCD and yCD-SAH relative activity against 5-FC where the wt-yCD activity set to 100%. The reaction mixture contained 10 mM 5-FC and 0.05 µM yCD in a buffer of 20 mM Tris, 100mM NaCl, pH 7.0. The reactions were carried out at 303 K in triplicates. The error bars are the standard deviation.
